# Lyme Disease: A Review with Emphasis on Latin America

**DOI:** 10.3390/microorganisms12020385

**Published:** 2024-02-13

**Authors:** Vanina Lucca, Sandra Nuñez, María Belen Pucheta, Nilda Radman, Teresita Rigonatto, Graciela Sánchez, Beatriz Del Curto, Dolores Oliva, Betina Mariño, Giuliana López, Serena Bonin, Giusto Trevisan, Nestor Oscar Stanchi

**Affiliations:** 1 Facultad de Ciencias Veterinarias, Universidad Nacional del Chaco Austral, Roque Sáenz Peña 3700, Argentinasandran@uncaus.edu.ar (S.N.); mpucheta@uncaus.edu.ar (M.B.P.);; 2 Facultad de Ciencias Veterinarias, Universidad Nacional del Noreste, Corrientes 3400, Argentina; trigonatto@vet.unne.edu.ar (T.R.);; 3 Facultad de Ciencias Veterinarias, Universidad Nacional de La Plata, La Plata 1900, Argentina; 4 Facultad de Ciencias Veterinarias, Universidad Nacional del Litoral, Esperanza 3080, Argentina; bmarino@fcv.unl.edu.ar; 5 Department of Medical Sciences, University of Trieste, 34100 Trieste, Italy; sbonin@units.it (S.B.); trevisan@units.it (G.T.)

**Keywords:** Lyme disease, *Borrelia burgdorferi*, Latin America

## Abstract

The spirochete *Borrelia burgdorferi* sensu lato (Lyme Group) is the causative agent of Lyme disease, transmitted to humans through tick bites carrying the bacteria. Common symptoms include fever, headache, fatigue, and the characteristic erythema migrans skin rash. If left untreated, the infection can affect joints, the cardiac system, and the nervous system. Diagnosis relies on symptoms, clinical signs (such as the rash), and potential exposure to infected ticks, with laboratory tests proving valuable when appropriately employed with validated methods. Most cases of Lyme disease respond effectively to a few weeks of antibiotic treatment. In Latin America, knowledge of Lyme disease is limited and often confounded, underscoring the significance of this review in aiding medical professionals in recognizing the disease. This study delves explicitly into Lyme disease in Argentina, neighboring countries, and other Latin American nations.

## 1. Introduction

Lyme disease, a multisystemic infectious disorder, is a systemic anthropozoonosis caused by the bite of ticks, usually from the Ixodidae family, that are infected with pathogenic spirochaetes of the *Borrelia* genus, that has emerged as a public health concern in various regions worldwide [1]. Its association with the *Borrelia burgdorferi* sensu lato (sl) (Lyme Group) has driven significant research efforts aimed at understanding its epidemiology, diagnosis, and treatment. While initially identified in areas of North America and Europe, Lyme disease has transcended geographic boundaries, especially in the northern hemisphere, and become a global challenge. In the Argentine context, the presence of this disease and its causative agent, *B. burgdorferi*, has been the subject of study and debate within the scientific and medical community [2,3].

Lyme disease is primarily transmitted through the bite of ticks belonging to the *Ixodes* genus, which act as vectors in transmitting *B. burgdorferi* sensu lato (sl) to humans and other hosts. As these ticks find new ecological niches due to climate and habitat changes, the geographical distribution of the disease has expanded significantly [4]. In Argentina, a nation known for its geographical and climatic diversity, the presence of different tick species and their potential to act as vectors for Lyme disease raise important questions.

The presence of *B. burgdorferi* sl and Lyme disease in Argentina constitutes a relevant and evolving topic that requires comprehensive scientific and medical attention.

The history of Lyme disease in Latin America is marked by a relatively recent recognition of its presence and increasing efforts to understand its epidemiology and clinical impact.

In the late 20th Century, Lyme disease was initially considered a regional concern, predominantly in North America and Europe. However, as awareness grew, cases began to be reported outside these traditional endemic regions. In the early 2000s, sporadic cases of Lyme disease were documented but not confirmed in Latin American countries, including Argentina, Brazil, Chile, and Mexico. These cases raised questions about the potential emergence of the disease in the region. The past decade has seen heightened research efforts to explore Lyme disease’s prevalence and impact in Latin America. Studies have investigated tick populations, identified potential vectors, and conducted serological analyses to detect *B. burgdorferi* antibodies in human and animal populations [5].

The diagnosis of Lyme disease in Latin America faces challenges due to its diverse clinical presentation, which can mimic other illnesses, and limited awareness among healthcare professionals. Additionally, the presence of other Borrelia species in the region adds complexity to diagnosis and classification.

In South America and the Caribbean, clinical and laboratory findings regarding *B. burgdorferi* sl infection have been documented but not confirmed in countries such as Argentina, Bolivia, Chile, Colombia, and Venezuela. Prevalence studies, attempts at isolation, and molecular detection of the agent have revealed serological evidence of the infection. However, confirmatory data are currently lacking. In Brazil, clinical, serological, and molecular studies have described a syndrome similar to or imitating Lyme disease [6].

Specifically, in Argentina, Mazzonelli et al. [7] found antibodies in dogs, and Stanchi-Balagué [2] detected antibodies in humans from rural areas through immunofluorescence. These studies ruled out cross-reactivity with other spirochetes through specific tests.

In Mexico and Cuba, Lyme disease has been reported, with the recent detection of *B. burgdorferi* sensu stricto (ss) DNA in ticks [5,8]. In addition, specific serological evidence has been confirmed through the detection of antibodies in patients with clinical and epidemiological suspicions of the disease.

Given the potential risk that the presence of the transmitting agent poses for Cuba, Herrera and colleagues [5] consider it important to review the topic in order to update specialists and other entities within the healthcare system that may be involved in the epidemiological surveillance and control of this disease.

While Lyme disease’s history in Latin America is relatively recent compared to other parts of the world, it is becoming increasingly recognized as an emerging health concern. Continued research, surveillance, and medical education are vital to understanding the disease’s dynamics, implementing effective preventive measures, and ensuring timely diagnosis and treatment. As the region’s awareness grows, collaborations between researchers, clinicians, and public health authorities play a crucial role in managing this complex and evolving public health challenge.

As cases of Lyme disease have been documented but not confirmed in various regions of Argentina, it is crucial to understand the dynamics of this disease and its potential to affect public health. By addressing these issues, we hope to contribute to a better understanding of the country’s current Lyme disease situation and provide a foundation for future research and strategies for prevention and control. This work aims to review and analyze the currently available information regarding the presence of *B. burgdorferi* and Lyme disease in Argentina and Latin America. Epidemiological findings, diagnostic methods employed, and relevant clinical and therapeutic considerations in the context of the Argentine population will be explored. Furthermore, the potential impact of environmental, geographical, and climatic factors on disease distribution and the interaction between hosts, ticks, and the bacterium will be evaluated.

## 2. Epidemiology of Lyme Disease with Special Reference to Latin America

### 2.1. Global Epidemiology

Lyme disease has a well-established epidemiology in North America, Europe, and Asia. The disease is primarily transmitted through the bite of infected Ixodes ticks, particularly *Ixodes scapularis* (black-legged tick) in North America and *I. ricinus* in Europe. The geographic distribution of Lyme disease is closely tied to the distribution of these tick vectors.

### 2.2. Latin American Context

In Latin America, the epidemiology of Lyme disease is still emerging and varies across different countries due to ecological, climatic, and geographical factors. The disease’s presence and distribution are influenced by the presence of suitable tick vectors, reservoir hosts, and interactions with human populations.

### 2.3. Key Aspects of Lyme Disease Epidemiology in Latin America

Tick Vectors: Different species of Ixodes ticks are found in various regions of Latin America. While *I. scapularis* is absent, species such as *I. pararicinus*, *I. loricatus*, and *I. aragaoi* have been considered potential tick vectors for *B. burgdorferi* in countries like Argentina and Brazil. Table 1 delineates the distribution of Borrelia species and their carriers across Central and South America.

Reservoir Hosts: Small mammals, birds, and other animals can serve as reservoir hosts for *B. burgdorferi*. Understanding the local reservoir hosts is essential for assessing the risk of transmission.

Binetruy and colleagues discovered that a novel Borrelia species, Candidatus *B. mahuryensis*, divergent from Lyme disease and Recurrent Fever Borrelia species, is prevalent in ticks associated with passerine birds in the tropical rainforests of French Guiana. Bird migration (for *Amblyomma longirostre*, *A. geayi*, and *A. maculatum*) and cattle transportation (for *A. maculatum*) emerged as two crucial factors influencing the distribution over long distances and across geographical barriers, thereby accounting for its wide geographic presence [9].

## 3. What Tick Species Are Likely to Transmit Lyme Disease in Argentina?

In Argentina, several tick species have been identified, and their potential involvement in the transmission of *B. burgdorferi* has been a topic of investigation. Among these tick species, *Ixodes pararicinus*, *I. loricatus*, and *I. aragaoi* have been proposed as potential vectors due to their similarities with known vectors in other regions and their presence in areas where cases of Lyme disease have been reported [10,11].

In Argentina, the tick species that could transmit this disease include:

*I. scapularis*: This species is the main carrier of Lyme disease in North America, but it has also been found in other parts of the world.

*I. pacificus*: This species is primarily found on the West Coast of North America, but it could also be present in other regions.

*I. ricinus* complex: This species is mainly found in Europe, but it has also been reported in other parts of the world (North Africa: Tunisia, Morocco). This species is the main transmitter of *B. burgdorferi* (sl) in Europe and Asia, but its presence has also been reported in Argentina, especially in the Patagonian region. This tick can infect humans, dogs, horses, sheep, cows, and other mammals, as well as birds and reptiles.

*I. pararicinus*, commonly found in the Southern Cone of South America, has been particularly interesting due to its ecological niche and potential for transmitting Lyme disease. This tick species shares habitat preferences with its North American counterparts and has been found on various wildlife hosts, raising concerns about its ability to transmit *B. burgdorferi* to humans.

Similarly, *I. loricatus*, distributed across different regions of Argentina, exhibits behaviors and habitats consistent with those of Lyme disease vectors. Its capacity to feed on multiple hosts and its interaction with various reservoir animals have raised questions about its potential role in the transmission cycle of *B. burgdorferi*.

*I. aragaoi*, although less studied, has also been considered as a possible vector in the region. Its presence in grassland ecosystems and interactions with a variety of hosts suggest a potential for involvement in the transmission of Lyme disease.

*A. cajennense*: This species is the most common and abundant in South America, ranging from the southern United States to northern Argentina. This tick can transmit several diseases, including Lyme borreliosis caused by *B. burgdorferi* ss (Baggio–Yoshinary Syndrome?). It can parasitize humans, horses, dogs, cats, pigs, rodents, and other mammals, as well as birds and reptiles.

*Rhipicephalus sanguineus*: This species is the most cosmopolitan of ticks and is found on all continents except Antarctica. This tick is the main vector of *B. burgdorferi* ss in North America and Africa, but its presence has also been detected in Argentina. Its preferred host is the domestic dog, but it can also infect humans, cats, rabbits, rodents, and other mammals.

Clinical and laboratory findings regarding *B. burgdorferi* sl infection have been documented in Latin America and the Caribbean in various countries such as Argentina, Bolivia, Chile, Colombia, and Venezuela. Studies on seroprevalence and the search for infections in humans and animals have been conducted, along with attempts at isolation and molecular detection of Borrelias. These efforts have provided serological evidence of infection, but unfortunately, in most countries, confirmation has not been achieved.

In Brazil, clinical, serological, and molecular studies have enabled the description of a syndrome similar to or imitating Lyme disease (Baggio–Yoshinari Syndrome). In Mexico, Lyme disease has been reported due to the recent detection of *B. burgdorferi* (ss) DNA in ticks. Additionally, specific serological evidence has been previously confirmed in patients with clinical and epidemiological suspicions of the disease [12,13,14]. *B. ibitipoquensis* has recently been isolated from *I. paranaensis* [14].

In Cuba, specific serological evidence suggesting infection has been found in different regions, such as in Pinar del Río, where 152 serum samples from individuals with clinical and epidemiological suspicions were examined. Furthermore, specific antibodies against *B. burgdorferi* (ss) have been detected in individuals from a community with a historical tick infestation [4,14,15].

Lyme disease occurs in individuals of all ages but is more frequent in men, with a significant increase in the rate observed [15].

Nonetheless, it is important to note that the identification of tick species as vectors for *B. burgdorferi* transmission requires comprehensive studies to confirm their ability to acquire, maintain, and transmit the pathogen. The complex interactions between ticks, the bacterium, and reservoir hosts need to be thoroughly investigated to establish the potential risk posed by different tick species in Argentina.

While the *Ixodes* genus remains a focus for potential Lyme disease vectors in Argentina, the specific tick species involved in transmitting *B. burgdorferi* in the region require further investigation. Understanding the biology, ecology, and behavior of these ticks is essential for assessing the risk of Lyme disease transmission and implementing appropriate preventive measures in areas where the disease poses a threat.

## 4. Scientific Background and Published Papers on Lyme Disease in Argentina, Neighboring Countries and Latin American Nations

**Figure 1:**
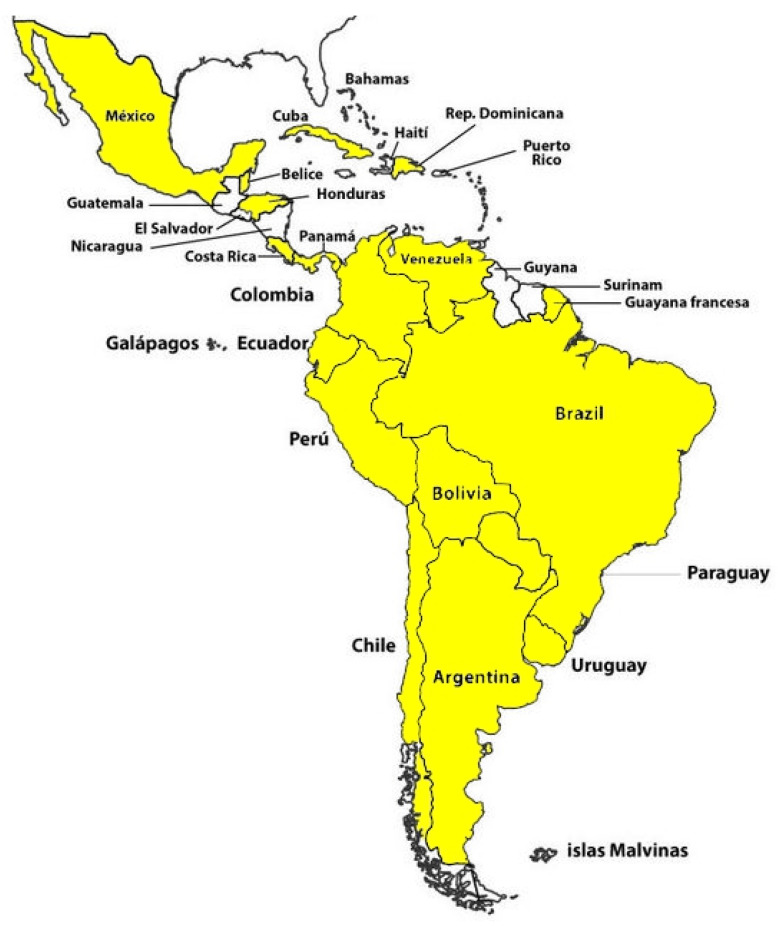
Latin American countries (highlighted in yellow) where potential cases of Lyme disease or the presence of infected ticks have been identified.

Here, we present a comprehensive overview of the scientific background and key research papers concerning Lyme disease in Argentina and neighboring countries, including Brazil, Uruguay, Paraguay, Bolivia, and Chile [16,17]. Additionally, we explore Lyme disease in Latin American countries such as Colombia, Costa Rica, Cuba, Ecuador, El Salvador, Guatemala, Honduras, Mexico, Nicaragua, Panama, Paraguay, Peru, Puerto Rico, the Dominican Republic, and Venezuela. Figure 1 illustrates the Latin American countries where potential cases of Lyme disease or infected ticks have been identified.

### 4.1. Argentina

In Argentina, Lyme disease has been a subject of increasing concern. Various studies have documented the presence of *B. burgdorferi* DNA in ticks collected from different regions. For instance, Nava et al. [10] conducted tick surveillance in multiple provinces and detected *B. burgdorferi* DNA in *I. pararicinus* ticks, highlighting its potential role as a vector. Nava described the presence of *B. burgdorferi* (sl) infecting ticks in Argentina for the first time. Unfed specimens of *I. pararicinus* collected from vegetation in Jujuy Province were tested for Borrelia infection by PCR targeting the flagellin (fla) gene, the rrfA-rrlB intergenic spacer region (IGS), and the 16S rDNA (rrs) gene.

Additionally, Lopes de Carvalho et al. [17] and Nava et al. [10] revealed the presence of *B. burgdorferi* (sl) in ticks collected from mammals in northwestern Argentina, providing further evidence of the pathogen’s circulation. On the other hand, Cicuttin [3] denies its presence in this country.

In the Argentine provinces of Chubut, Río Negro, and Santa Cruz [18], rodents infected with species from the *B. burgdorferi* complex were found in nymphal states of *I. neuquenensis* in the province of Río Negro and *I. sigelos* in the provinces of Chubut and Santa Cruz. However, the pathogenic relevance of these Borrelias remains unknown.

In the provinces of Entre Ríos, Misiones, Formosa, Salta, Córdoba, San Luis, and Buenos Aires [3], from the 422 specimens of the species *A. aureolatum*, *A. brasiliense*, *A. ovale*, *A. sculptum*, *A. tigrinum*, and *A. tonelliae* analyzed, all samples were negative.

In the Yungas biogeographic region of Argentina, the tick species infesting cattle and humans along an altitudinal gradient were determined. In the provinces of Jujuy, Salta, and Tucumán [11], the presence of Borrelias in populations of *I. pararicinus* ticks collected from the environment and birds was investigated [10]. They found Borrelia genospecies from the *B. burgdorferi* complex in nymphs collected from birds of the Turdidae family. In Tucumán, one nymph was collected from a bird of the Furnariidae family in Jujuy, and two females were collected from vegetation in Salta. However, the *Borrelia* genospecies found by these authors are not related to those currently recognized as human pathogens.

In a comprehensive study conducted by Cicuttin and colleagues, epidemiological aspects of *Borrelia* spp. were investigated in an urban area, presenting the first report of *Borrelia* presence in *A. aureolatum*. The authors collected ticks from vegetation, birds, and dogs. One hundred and twenty ticks were collected from 47 birds and identified as *I. auritulus* and *A. aureolatum*. Additionally, 1090 ticks collected from vegetation, 100 from birds, and 89 from dogs were analyzed for Borrelia infection using PCR tests targeting the flagellin (fla) and 16S rRNA genes. Four *I. auritulus* nymphs (0.7%) collected from vegetation were positive. Five *A. aureolatum* nymphs (45.4%) and five groups of larvae (minimum infection rate 13.5%), eighteen nymphs (40.9%) and one female (14.3%) of *I. auritulus* collected from birds were also positive. The remaining samples were negative. The authors highlighted that the phylogenetic tree generated with *fla* sequences showed that seven of the eight different *Borrelia* haplotypes detected in *I. auritulus* formed an independent lineage within the *B. burgdorferi* (sl) complex, along with *Borrelia* sp. sequences detected in *I. auritulus* from Canada and Uruguay. The epidemiological risk of the *Borrelia* genospecies associated with *I. auritulus* appears to be low because this tick is not aggressive towards humans, but it aids in maintaining borrelial spirochetes in enzootic transmission cycles. The pathogenicity towards humans of Borrelia found in *A. aureolatum* is unknown; however, adults of this tick species are known to bite humans [19].

### 4.2. Lyme Disease Research in Argentina: Serological Analyses in Humans and Dogs

In Argentina, the exploration of Lyme disease has extended beyond tick surveillance to include serological analyses in both humans and animals, shedding light on the potential exposure to *B. burgdorferi*.

### 4.3. Lyme Disease Research in Argentina: Serological Studies in Humans

Stanchi and Balagué [2] conducted a pioneering serological study aimed at assessing the exposure of individuals to *B. burgdorferi*. They analyzed serum samples from individuals residing in regions with reported tick activity. The study employed indirect immunofluorescence to detect antibodies against *B. burgdorferi* antigens. This study conducted additional tests to verify the absence of cross-reactivity with *Leptospira* sp. (Microscopic Agglutination Test) and *Treponema pallidum* (with Venereal Disease Research Laboratory antigen -VDRL-). Complementary studies were undertaken with Rheumatoid Factor (RF), C-reactive Protein (CRP), and anti-streptolysin O (ASTO). The results indicated seropositivity in a subset of the population, suggesting past exposure to the pathogen. While this initial study provided valuable insights into the potential human exposure, further investigations are needed to understand the extent and distribution of Lyme disease in Argentina.

### 4.4. Lyme Disease Research in Argentina: Serological Studies in Dogs

Mazzonelli and Brihuega [7] focused their research on the seroprevalence of *B. burgdorferi* in dogs, recognizing them as sentinel animals due to their close interaction with ticks. Using serological assays similar to those employed in human studies, the researchers assessed serum samples from dogs across various regions. Their findings revealed evidence of exposure to *B. burgdorferi* in dogs, indicating the potential circulation of the pathogen in tick populations. This study underscored the importance of considering domestic animals in disease surveillance efforts.

Building on these initial serological studies, subsequent research efforts have aimed to expand the understanding of Lyme disease in Argentina. Investigations have delved into the diversity of tick species, the presence of *B. burgdorferi* DNA in ticks, and the clinical presentation of suspected Lyme disease cases. While not all studies have reported conclusive evidence of the pathogen’s prevalence, the collective body of research highlights the need for continued vigilance and comprehensive studies.

Lyme disease research in Argentina faces challenges such as the diversity of tick species, the potential presence of related Borrelia species, and the varying environmental factors that influence disease transmission. Collaborative efforts between researchers, clinicians, and public health authorities are essential to develop a comprehensive understanding of the disease’s epidemiology, clinical manifestations, and distribution. Serological analyses in both humans and dogs have contributed to the exploration of Lyme disease in Argentina. Studies conducted by Stanchi and Balagué as well as Mazzonelli and Brihuega have provided valuable insights into the exposure of individuals and animals to *B. burgdorferi*. As the research landscape evolves, continued investigations are necessary to address the complex dynamics of Lyme disease in the region and guide effective prevention and control strategies.

### 4.5. Brazil

In Brazil, Lyme disease research has been centered on elucidating the tick fauna and their potential role in transmitting *B. burgdorferi*. Fernando Aguilar López [11] found molecular evidence of *B. burgdorferi* sl in patients from central–western Brazil. Additionally, the work of Luz et al. identified *B. burgdorferi* in ticks collected from birds, suggesting a potential avian reservoir for the pathogen [20]. In 2020, Candidatus *Borrelia ibitipoquensis* was identified in Brazil, transmitted by *I. paranaensis*, and is related to the *Borrelia valaisiana* genospecies [14].

Mantovani and colleagues describe a Lyme disease-like syndrome (LDLS) in Brazil that seems to be a new tick-borne disease found in Brazil, which is confused with the LD described in the Northern Hemisphere. This syndrome has grown significantly and is becoming a serious public health problem. The condition is considered to be a zoonosis transmitted by ticks of the genus *Amblyomma*. Although PCR analyses were negative, direct observations revealed spirochetes in the blood [21].

Furthermore, in 2020, Jorge and colleagues investigated *Borrelia* infection among vampire bats (*Desmodus rotundus*) in the semi-arid region of Brazil. Molecular analysis of the bats’ organs identified haplotypes of a new Borrelia organism in nearly all organs in 5% of the bats. Phylogenetic analysis of the borrelial genes *rrs* and *flaB* indicated that the *Borrelia* sp. associated with the vampire bat in this study forms a monophyletic group with a Borrelia recently reported to be associated with a bat from Colombia. This Borrelia species is distinct from the three main groups currently recognized: the relapsing fever group (RFG), the *B. burgdorferi* (sl) group, and the reptile–monotreme group [22].

### 4.6. Uruguay

Lyme disease research in Uruguay has been relatively limited, but studies have indicated the presence of *Ixodes* spp. ticks, which could be potential vectors. While the prevalence of *B. burgdorferi* remains to be fully understood, studies like that of Venzal et al. [23] have highlighted the diversity of tick species in the country, laying the groundwork for further investigation.

### 4.7. Paraguay

Paraguay’s contribution to Lyme disease research has been minimal, with few published studies on the topic. The country’s geographical proximity to endemic regions raises questions about the potential for pathogen transmission. Given the lack of comprehensive studies, further research is needed to assess the risk of Lyme disease in Paraguay [5].

### 4.8. Bolivia

Bolivia’s diverse ecosystems offer an intriguing context for Lyme disease research. The potential interaction between these ticks and *B. burgdorferi*, however, requires more investigation to assess the risk to human and animal health. Ciceroni et al. [24] report of the presence of Lyme borreliosis and tick-borne relapsing fever in Bolivia. For Lyme borreliosis, these findings represent further evidence to support its existence in South America. Briaçon Ayo [16] mentions that there are confirmed disease cases in this country.

### 4.9. Chile

Gonzalo Osorio [25] searched for *B. burgdorferi* in ticks using PCR but did not find positive results. Chile has seen emerging interest in Lyme disease research, with studies focusing on tick surveillance and detecting *B. burgdorferi* DNA in ticks collected from various hosts. Additionally, cases of Lyme disease in humans and dogs have been reported, sparking discussions about its endemicity in certain regions.

### 4.10. Perú

Cervantes [26] reports the presence of clinical cases of the disease, although he does not confirm its presence.

### 4.11. Colombia

Mancilla-Agrono [27] reports the first molecular evidence of the presence of *B. burgdorferi* ss in South America. On the other hand, López and colleagues investigated 205 bats captured in six municipalities of Córdoba department, Colombia, describing the first molecular evidence of *Borrelia* spp. in that region, highlighting that several bat species harbor Borrelia spirochetes [28].

### 4.12. French Guiana

In French Guiana, several suspected cases were investigated through a committee established to determine the presence of Lyme disease. Twenty-six patients were included. The diagnosis of Lyme disease was confirmed in three patients (11%) with early localized Lyme disease, presumably acquired in French Guiana, but with no confirmation of the acquisition of Lyme disease in that place [29].

### 4.13. Belize

A study by Cline (2016) detected antibodies to *B. burgdorferi* (sl), the main cause of Lyme disease in North America, in 4.8% of field-collected ticks from the Cayo District of Belize. However, the study did not confirm the presence of the bacteria in the ticks by molecular methods, and the clinical significance of these findings is unclear [30].

### 4.14. Costa Rica

Villalobos-Zúñiga and Somogyi reported in 2012 a native case of Lyme disease in Costa Rica; however, the diagnosis was performed using immunofluorescence, so it could be attributed to cross-reactions as they did not carry out either ELISA or WB. On the other hand, in a study conducted by Montenegro et al., only one dog tested positive for *B. burgdorferi* sl [31,32].

### 4.15. Honduras

A study conducted on wild cats in Honduras found positivity for Lyme Borrelia and other pathogens in these animals [33]. Robles and colleagues (2018) reported a case of Lyme disease in Honduras with positive results from confirmatory ELISA and Western Blot tests in 2004 [34]. And, more recently, Aguilar Andino (2022) presented another case of neuroborreliosis in Honduras [35].

### 4.16. Panama

In a molecular study on *I. boliviensis* and *I. tapirus* in Panama, the presence of three genotypes of the *B. burgdorferi* (sl) complex was confirmed in 4 out of 27 ticks [36].

### 4.17. Dominican Republic

Castillo Ariza in 1993 described an imported clinical case of Lyme disease in the Dominican Republic. More recently, in a thesis study conducted by Zaglul-Ricardo and Acosta Díaz (2021) in the Dominican Republic, during their investigation on Pediatric Autoimmune Neuropsychiatric Disorders Associated with Streptococcal Infections (PANDAS), they found that 8% of the children tested positive for Lyme disease [37,38].

### 4.18. Ecuador

McCown and Monterroso (2016) found no evidence of this disease in an epidemiological study of 100 canines in the cities of Manta and Guayaquil. On the other hand, Alarcón-Guzmán et al. (2019) described the first case of Lyme disease in a 12-year-old child in Ecuador through positive ELISA and Western Blot tests [39].

In Mexico, Constantino Salazar et al. discuss the current perception of Lyme disease as exotic in the country. However, their assessment of the potential risk for *B. burgdorferi* transmission in Mexico identified regions with the highest potential of becoming endemic areas of Lyme disease. This was attributed to the arrival of hard ticks and birds infected by *B. burgdorferi* [40]. Despite Lyme disease not being officially reported, clinical and epidemiological suspicions, coupled with serological evidence suggestive of *B. burgdorferi* (sl) infection, provide the first confirmatory evidence of an endemic case of Lyme disease in Mexico. The analysis considered risk factors for the human population, the diversity of Borrelia species, and their geographic distribution. Six Borrelia species were reported in a total of 1347 cases, with 398 involving humans. The study highlighted that women and children from rural communities were more susceptible to Lyme borreliosis [41]. This study also marks the first report identifying coinfection of *Leptospira interrogans* and *B. burgdorferi* sl in wild rodents, including *Heteromys irroratus* and *Sigmodon hispidus* in Nuevo Leon, and *Heteromys gaumeri* in Quintana Roo, Mexico. These infected wild rodent species pose a risk factor for the exposed population in Mexico’s sylvatic and rural areas [42]. Despite Mexico City not being reported as an endemic area, we present a case involving a 29-year-old female patient originally from Mexico City. She developed symptoms starting with pain in the second finger of her left hand after a visit to the Aragon forest in Mexico City. A Western Blot test yielded a positive result for *B. burgdorferi*, establishing the definitive diagnosis of Lyme borreliosis [43].

### 4.19. Cuba

Rodriguez and colleagues report on two possible cases of Lyme disease in Cuba. Although not previously reported in the country, the existence of the disease has been suspected for some years. These cases, involving individuals bitten by ticks and exhibiting signs and symptoms compatible with Lyme disease according to the literature, were serologically confirmed using various laboratory techniques, including indirect immunofluorescence, ELISA, and Western Blotting. The results suggest the presence of this borreliosis in Cuba [44].

### 4.20. Venezuela

Sánchez and colleagues conducted a review of the medical history of an infant from a rural area (Palmira, Táchira State) who presented with erythema migrans, fever, anorexia, and lymphadenopathy following a tick bite from the Ixodes genus (*Amblyoma americanum*). Serological testing for *B. burgdorferi* was performed to determine the presence of Lyme disease in Venezuela [45]. Espinoza-León and colleagues found no association between localized scleroderma lesions and *B. burgdorferi* in Venezuelan patients [46]. Results from Arocha Sandoval and colleagues demonstrate the presence of antibodies against *B. burgdorferi* in a sample of the population of Zulia State, both in suspected patients and asymptomatic individuals, using ELISA. These findings pave the way for definitive diagnosis through more specific tests such as immunoblot for Lyme disease in Venezuela [47].

### 4.21. El Salvador, Nicaragua, Haiti, Puerto Rico, and Guatemala

Lyme disease has not been detected [48,49,50]. In an extensive literature review, Faccini-Martínez and colleagues (2022) found no information regarding the existence of the disease in El Salvador, Nicaragua, Haiti, Puerto Rico, and Guatemala. Additionally, they reflect that the current diagnosis of Borrelia fever group infections remains deficient, with overlooked cases, and the impact of spirochetes on animals and humans is not fully understood. The authors also thoroughly analyze the history and potential vectors of tick-borne diseases [51].

## 5. Conclusions

In reviewing the existing literature, we observe that numerous authors have explored the potential presence of *Borrelia burgdorferi* (sl) using molecular biology techniques in Ixodid ticks in Latin America, including the Argentine Republic. While ticks of the genus Ixodes may not be significant human parasites in Latin America, those belonging to the genus *Amblyomma* hold relevance.

From the studies conducted in Argentina and Latin America, it becomes evident that there are high probability of Lyme disease being present in this region. However, conclusive evidence is still lacking, necessitating confirmatory studies through PCR and the causative agent’s isolation. Moreover, it is imperative to identify the specific tick species responsible for transmitting the disease.

Furthermore, birds may play a role in distributing or disseminating this microorganism, transcending geographical barriers and distances, potentially contributing to the emergence of Lyme disease and other borrelioses in various countries.

To address these knowledge gaps, robust research policies for Lyme disease should be implemented to provide a definitive answer regarding its potential presence in South America and Latin American nations. Comprehensive studies that achieve the isolation of the microorganism or its detection in its vector and host by PCR are crucial to deepen our understanding of Lyme disease dynamics in this region.

## Figures and Tables

**Table 1:** Geographic distribution of Borrelia species and their carriers in Central and South America.

Geographic Area	Country	Hard Tick	Carrier	Borrelia Species Pathogenic to Humans
Central America	Mexico	*Ixodes kingi* *Ixodes hearley* *Ixodes scapularis*	*Microtus mexicanus* *Neotoma mexicana* *Neotomodon alstonio* *Peromyscus leucopus* *Peromyscus maniculatus* *Geothlypis trichas*	*Borrelia burgdorferi *ss
	Costa Rica	*Ixodes affinis*		*Borrelia burgdorferi* sl
	Panama	*Ixodes boliviensis* *Ixodes affinis*		*Borrelia burgdorferi* sl
	Dominican Republic	*Ixodes pararicinus*		*Borrelia bissettii* *B. burgdorferi* sl
South America	Brazil	*Ixodes longiscutatus* *Ixodes paranaensis* *Amblyomma cajennense* (?)	*Rodents* *Streptoprocne biscutata*	*Borrelia* sp. *Aplotype Pampa candidatus Borrelia ibitipoquensis*
	Argentina	*Ixodes pararicinus* *Ixodes affinis*	Turdus Birds	*Borrelia burgdorferi* sl
	Uruguay	*Ixodes aragaoi* *Ixodes auritulus*	Rodents Passerinc Birds	*Borrelia bissettii*
